# The effect of inbreeding, body size and morphology on health in dog breeds

**DOI:** 10.1186/s40575-021-00111-4

**Published:** 2021-12-02

**Authors:** Danika Bannasch, Thomas Famula, Jonas Donner, Heidi Anderson, Leena Honkanen, Kevin Batcher, Noa Safra, Sara Thomasy, Robert Rebhun

**Affiliations:** 1 Department of Population Health and Reproduction, School of Veterinary Medicine, University of California-Davis, Davis, CA 95616 USA; 2 Department of Animal Science, University of California-Davis, Davis, CA 95616 USA; 3 Wisdom Health Genetics, Kinship, FI-00290 Helsinki, Finland; 4 Zoetis, Parsippany, NJ USA; 5 Department of Surgical and Radiological Sciences, School of Veterinary Medicine, University of California-Davis, Davis, CA 95616 USA; 6 Department of Ophthalmology & Vision Science, School of Medicine, University of California-Davis, Davis, CA 95616 USA

**Keywords:** Canine, Genetic, Inherited, Morbidity, Mortality

## Abstract

**Background:**

Dog breeds are known for their distinctive body shape, size, coat color, head type and behaviors, features that are relatively similar across members of a breed. Unfortunately, dog breeds are also characterized by distinct predispositions to disease. We explored the relationships between inbreeding, morphology and health using genotype based inbreeding estimates, body weight and insurance data for morbidity.

**Results:**

The average inbreeding based on genotype across 227 breeds was F_adj_ = 0.249 (95% CI 0.235–0.263). There were significant differences in morbidity between breeds with low and high inbreeding (H = 16.49, *P* = 0.0004). There was also a significant difference in morbidity between brachycephalic breeds and non-brachycephalic breeds (*P* = 0.0048) and between functionally distinct groups of breeds (H = 14.95 *P* < 0.0001). Morbidity was modeled using robust regression analysis and both body weight (*P* < 0.0001) and inbreeding (*P* = 0.013) were significant (*r*^*2*^ = 0.77). Smaller less inbred breeds were healthier than larger more inbred breeds.

**Conclusions:**

In this study, body size and inbreeding along with deleterious morphologies contributed to increases in necessary health care in dogs.

**Supplementary Information:**

The online version contains supplementary material available at 10.1186/s40575-021-00111-4.

## Background

Most modern dog breeds were established within the last 200 years [1]. Dog breeds can be characterized by small numbers of founders, with strong selection for morphology, size and color. In addition, population bottlenecks due to historical events, and the introduction of closed studbooks in the last 100 years has shaped the formation of modern dog breeds [2]. These factors have all contributed to high levels of inbreeding within breeds. The level of inbreeding can be estimated using pedigrees, which were commonly used to determine the coefficient of inbreeding (F_*p*_) within individuals and breeds [3]; however, unless calculations include the complete pedigree [4, 5], F_*p*_ may underrepresent the actual inbreeding by 5–10 fold [6–8]. More recently, measurement of genetic inbreeding levels through direct genotype-based methods has become a feasible option. Such approaches to determine inbreeding have provided evidence that dog breeds [4, 5, 8, 9] have levels of inbreeding that are considered extremely high (> 0.1) in other species and where the effects of inbreeding depression are expected to occur [10–12].

There is evidence that high levels of inbreeding have consequences on health. Close inbreeding has a negative effect on litter size and neonatal survival [13]. Domestication and breed formation have unintentionally increased the number of deleterious genetic variants within breeds [14, 15]. Individual breeds also have strong predispositions to specific inherited diseases [16–19]. The Online Mendelian Inheritance in Animals website (OMIA, April 2021, omia.org) currently catalogues 796 canine disorders and traits [20]. Some of these have been found to be due to high allele frequencies of deleterious recessive mutations (for example [21, 22]), while others have remained elusive and are likely polygenic in nature [23, 24]. Some of the breed disease predispositions may be due to concentrated genetic disease polymorphisms rather than segregating traits [25, 26]. Consistent with this theory, mixed breeds have lower risk of diseases than purebreds [27]; however, the risk can vary by disease [28, 29] and has not been evaluated by level of inbreeding.

Body size and morphology vary within and between breeds and have an effect on lifespan in dogs. Breed average lifespan is inversely correlated with breed average body weight [30], with smaller dogs living longer than larger dogs. However, lifespan differs between breeds of the same weight class indicating that more than body size is a factor [31–33]. Morphological characteristics of dog breeds could be one factor, since it can have a critical effect on their lifespan and health [18, 34]. Results for the effect of inbreeding on mortality have varied between previous studies [9, 32].

Overall health comparisons across breeds have been challenging to perform since individual measurements are not available. In addition to the examples cited in the previous paragraph, there are numerous examples in the canine literature of the utilization of breed average measures for height, weight and health for successful genetic association studies [35–43]. In some examples, breed-average measures resulted in a clear increase in GWAS power for detecting body size associations (compared to individual phenotypes) [40].

We utilize complied pet insurance data reported relative to number of years insured to quantify relative health across dog breeds. Breed-based morbidity measures (non-routine veterinary care events) were compared to breed morphology, body size and estimates of inbreeding based on genotypes. High levels of inbreeding were identified across breeds as expected. Robust regression analysis identified statistically significant effects of both inbreeding and body size on morbidity across breeds.

## Results

A large dataset (227 breeds; dataset 1) of median heterozygosity values (H) was obtained through commercial DNA testing of 49,378 dogs. The mean number of individuals per breed was 217.5 (95% Confidence Interval 162–273.1) and the range of individual dogs/breed was 30–4728. Since the H values do not provide a simple relationship to pedigree, we wanted to determine what their equivalency was to the coefficient of inbreeding (F). The genotype-based coefficient of inbreeding was determined in 19 breeds with 8–20 individuals in each breed (dataset 2) for comparison to dataset 1. Dreger at al [8] used 10 individuals per breed and calculated F based on genotypes and the correlation with dataset 2 was 0.85 (12 breeds in common). Yordy et al. [9] used 6–724 individuals per breed and the correlation with dataset 2 was 0.75 (15 breeds in common). F from dataset 2 was highly correlated with H from dataset 1 (Pearson correlation coefficient of − 0.8899 (*P* < 0.0001) for the same 19 breeds. Linear regression (*r*^*2*^ = 0.79) was performed to obtain adjusted F values (F_adj_) for the dataset 1 breeds. Based upon linear regression, an H value of 33.1% was equivalent to F of 0.25. The H values were adjusted based on the regression to provide breed estimates of inbreeding for the 227 breeds in dataset 1 (F_adj_). These adjusted values from dataset 1 were compared to adjusted values from [9] which included 97 breeds in common and the Pearson correlation was 0.883 and [8] which included 75 breeds in common and was 0.89.

**Fig. 1:**
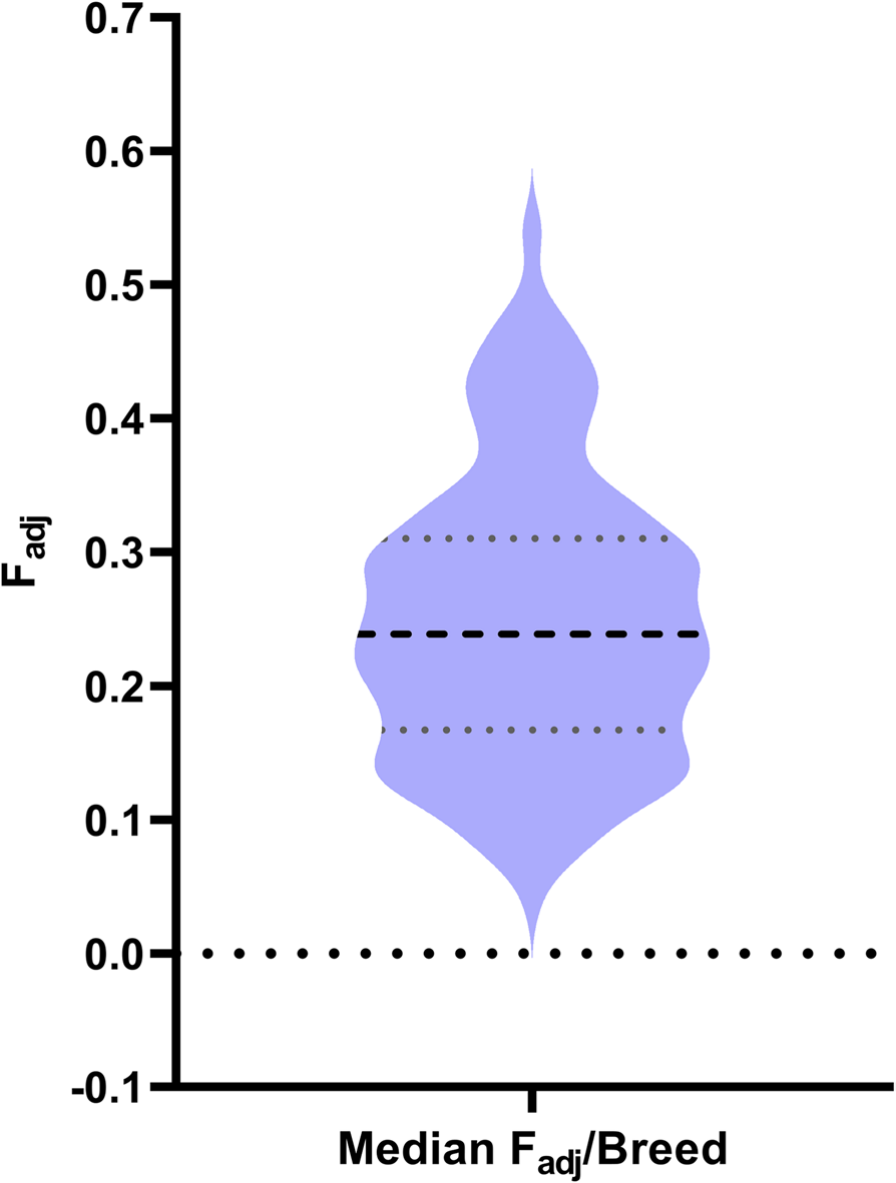
Violin plot of Median F adjusted values across breeds. The percentage heterozygosity for 227 breeds or varieties was adjusted to an estimate of Wrights inbreeding coefficient-F_adj_. Median and quartiles lines are indicated

The mean of the F_adj_ values for 227 breeds was 0.249 (95% CI 0.235–0.263) (Fig. 1). Strikingly few breeds (*N* = 12) had low inbreeding values (< 0.10). The breeds with the lowest levels of inbreeding were mostly landrace breeds or breeds with recent cross breeding. To put the inbreeding values in context, the breeding of two first cousins produces F = 0.0625, two half siblings F = 0.125 and two full siblings or parent-offspring F = 0.25.

**Fig. 2:**
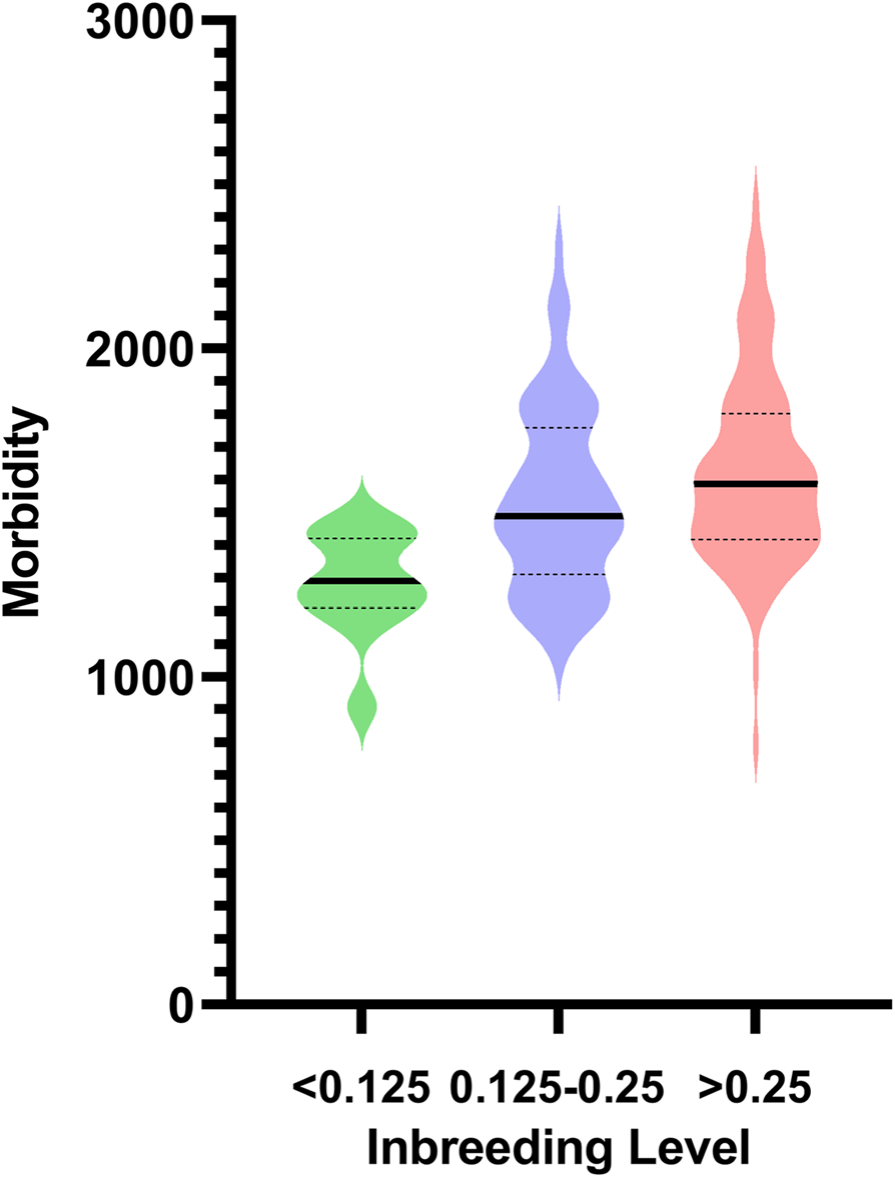
Morbidity by inbreeding levels. Morbidity values as VCE/10,000 dog years at risk (95% confidence intervals and mean are shown) plotted by mean F_adj_ within each breed in relevant categories. There were 11 breeds with F_adj_ < 0.125; mean morbidity for those breeds was 1282 (95% CI 1173–1391). For F_adj_ between 0.125 and 0.25, there were 66 breeds with a mean of 1537 (95% CI 1468–1606), and for F_adj_ over 0.25 there were 85 breeds with a mean of 1626 (95% CI 1562–1690). Significant differences for morbidity were identified between the three categories of F_adj_ using Kruskal-Wallis test (*P* = 0.0003). The relative risk morbidity compared to mixed breed dogs (F = 0 in dataset 1 and F_adj_ = 0.037 in dataset 2) for low inbred dogs was 1.01, meaning that they have a 1% increase in veterinary care events compared to mixed breed dogs. Breeds with inbreeding between 0.126 and 0.25 had a relative risk of 1.22 i.e. a 22% increase in veterinary care events while breeds with inbreeding over 0.25 had a relative risk of 1.29 i.e. a 29% increase in veterinary care events compared to mixed breed dogs

In order to investigate the effect of inbreeding level on health we utilized breed-based health data from Agria pet insurance. Morbidity values were available for 162 of the breeds with F_adj_ values. The mean morbidity across all breeds was 1574 (95% CI 1527–1622) per 10,000 DYAR (Dog Year at Risk) and for mixed breeds it was 1265. Morbidity was compared between three different categories of inbreeding (Fig. 2).

**Fig. 3:**
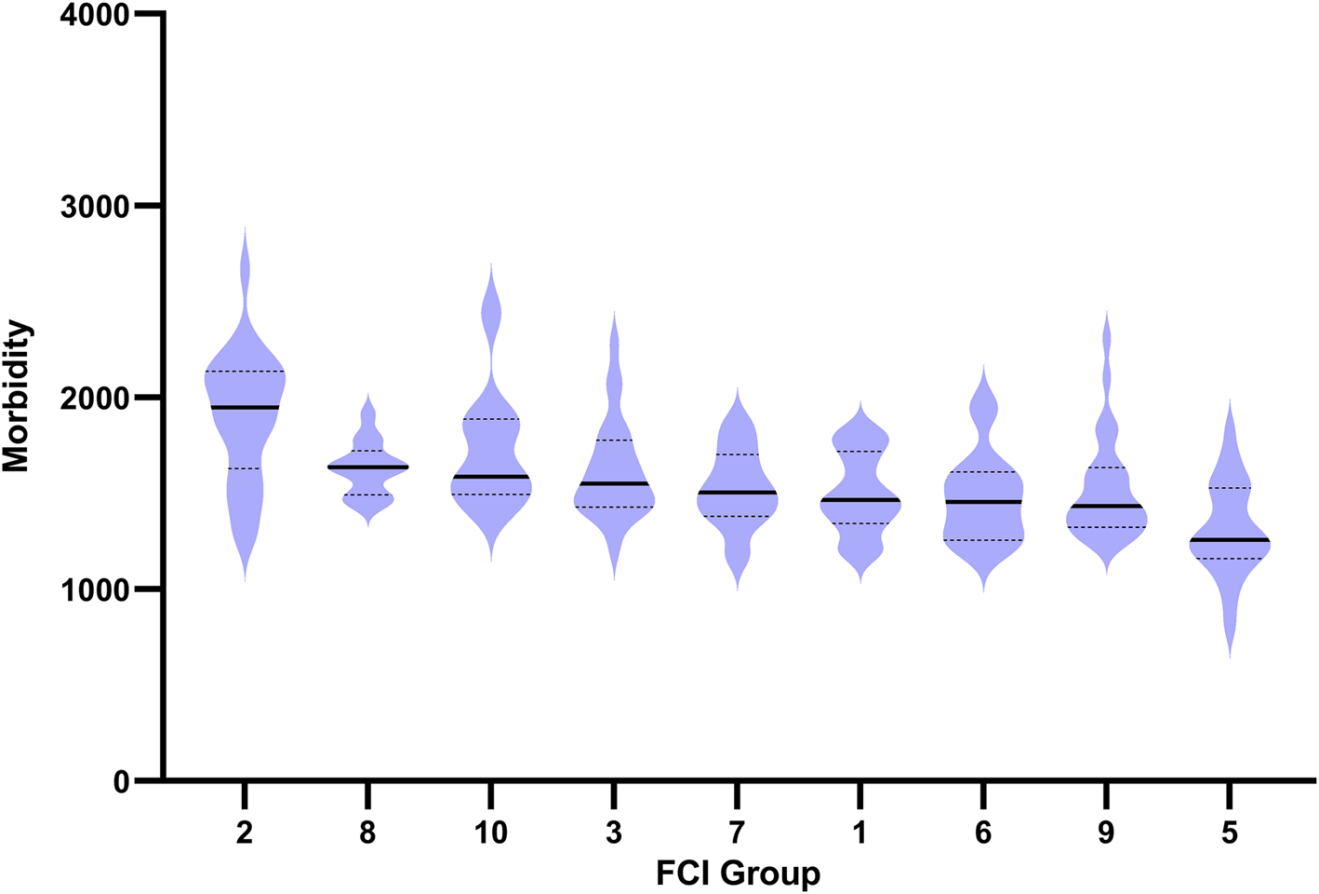
Morbidity values within FCI groups. Violin plots of morbidity as number of veterinary care events/10,000 dog years at risk plotted by FCI group (95% Confidence intervals and mean are shown). FCI breed groupings are ordered from high mean morbidity on the left to low mean morbidity on the right. 2 = pinscher, schnauzer, molossoid, swiss mountain and cattle dogs, 8 = retrievers, 10 = sighthounds, 3 = terriers, 7 = pointing dogs, 1 = sheepdogs and cattledogs, 6 = scent hounds and dachshunds, 9 = companion and toy, and 5 = spitz and primitive types

Breeds were evaluated based on their FCI (Fédération Cynologique Internationale) groups as a means of uncovering differences in health and inbreeding between breed types. There was no significant difference between FCI groups for inbreeding F_adj_ (*P* = 0.06), however there were significant differences between groups for morbidity (Kruskal-Wallis, *P* < 0.0001) (Fig. 3). Most notably, FCI group 5 composed of primitive breeds had low mean morbidity (1308) and group 2 which includes the Molossian/Mastiff types had a very high mean morbidity (1897) (a 45% increase). It should be noted that both body size and morphology can vary within most of these groups.

Breed morphology was evaluated using the Mann Whitney *U* test to determine if there were differences in morbidity between morphological breed groupings. Brachycephalic breeds had significantly higher morbidity and median inbreeding than non-brachycephalic breeds, but lower median body weight (Table 1).

**Table 1:** Comparison of Brachycepahlic and non-brachycephalic breeds

	Non-Brachycephalic	Brachycephalic	*P* value
Morbidity Median/10000 DYAR	1508 (*N* = 148)	1829 (*N* = 14)	0.0048
F_adj_	0.2450	0.319	0.0025
Body weight	18.26	6.69	0.0262

In order to evaluate the effects of body weight and inbreeding on morbidity in dogs, Spearman correlations were performed. Spearman r between morbidity and F_adj_ was 0.29, *P* = 0.0002 and between morbidity and body weight it was 0.41, *p* < 0.0001. Recognizing that there was a complex relationship we developed a model to predict morbidity across breeds which included body weight and median inbreeding. Brachycephalic breeds were excluded since their conformation so heavily influenced their health leaving 148 breeds with morbidity, inbreeding and body size data.

**Fig. 4:**
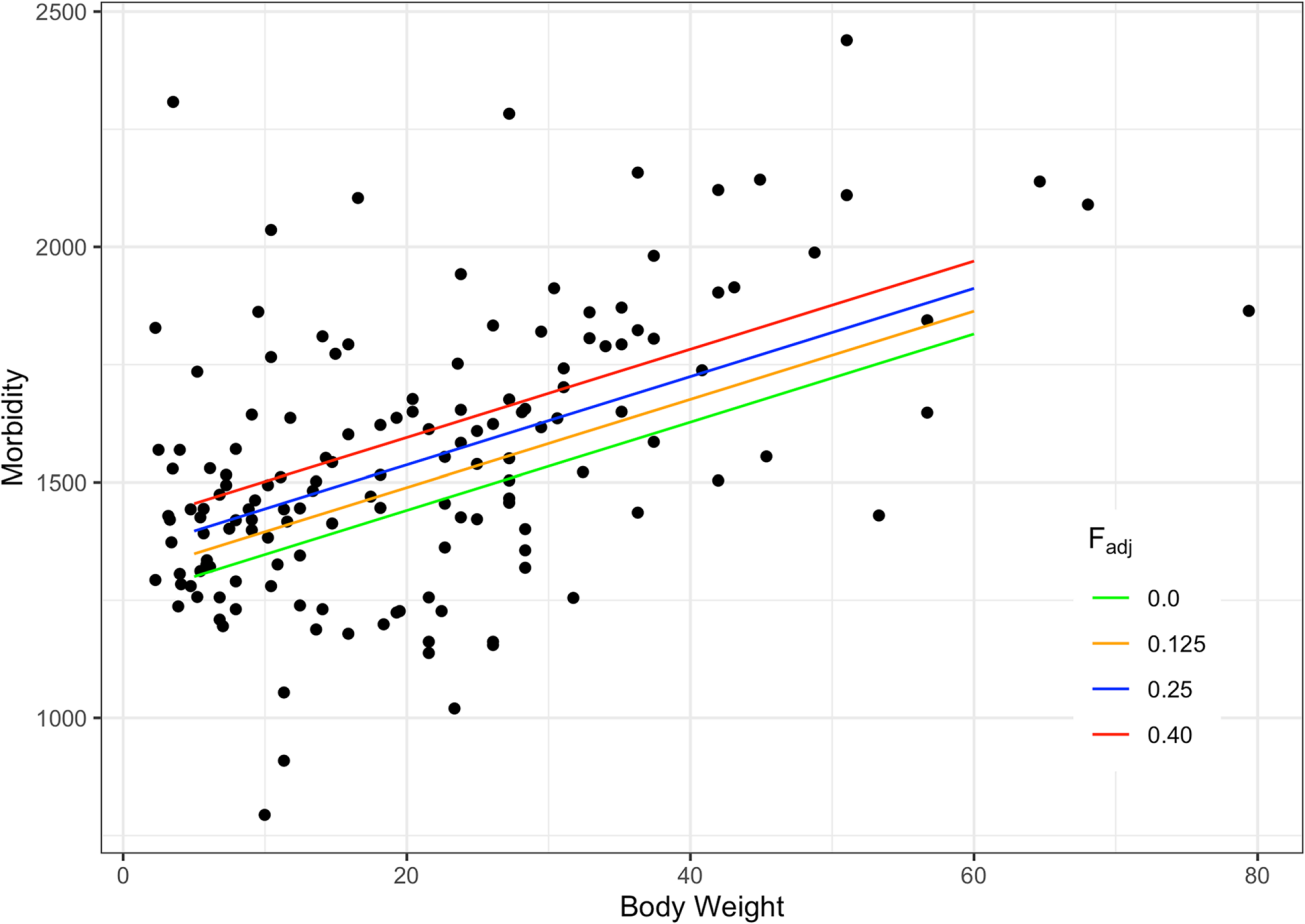
Morbidity as a function of body weight and inbreeding. Morbidity in number of veterinary care events /10,000 dog years at risk for breeds plotted by mid-range body weight. Regression lines are shown for different levels of inbreeding as indicated. As an example, the morbidity values for a 30 kg dog at no inbreeding (F_adj_ = 0) are predicted to be 1534.5 (SE 36.75) compared to high inbreeding (F_adj_ = 0.40) 1688 (SE 24.47) which is a 10% increase in veterinary care events. The morbidity value for a 5 kg dog at F = 0.25 is 1396.9 (SE 18.88) and for a 60 kg dog it is 1911.8 (SE 38.67), which is a 37% increase in veterinary care events

Parameter estimates for the robust regression analysis are shown in Table 2. The most striking element of the table is the significant difference that both F_adj_ and body weight appear to exert on morbidity across breeds. The *r*^*2*^ for this model is 0.77. In order to put these results in context, we have graphed morbidity versus body weight and added regression lines for the effect of different inbreeding levels (Fig. 4).

**Table 2:** Parameter estimates for the analysis of morbidity in a model with regressions on body weight and inbreeding

Parameter	Estimate	Std Error	*P* value
Intercept	1618.6	115.48	< 0.0001
F_adj_ *(10%)*	38.69	3.2	0.013
Body Weight *(1 kg)*	9.36	1.07	< 0.0001

## Discussion

Breed average measures of inbreeding, body weight and cumulative insurance data on morbidity were used to identify a relationship between body size, body morphology, inbreeding and health in dogs. Breeds with higher inbreeding levels required greater amounts of veterinary care as did brachycephalic breeds and there were significant differences in required health care between FCI breed groupings. We identified a significant effect of both body size and inbreeding on morbidity across dog breeds with larger sized and more inbred breeds receiving more veterinary care throughout their lives.

The inbreeding values within dog breeds were very high, with the mean being 0.24, just below the coefficient of inbreeding obtained from breeding full siblings. The breeds with low inbreeding included recent cross breeds (Tamaskan Dog, Barbet and Australian Labradoodle) and landrace breeds (Danish-Swedish Farmdog, Mudi and Koolie), supporting the notion that high inbreeding is a result of closed stud books or small numbers of founders or both. It also demonstrates that it is possible to have consistent breed type without inbreeding.

Similar to another recent study, brachycephalic dogs require more veterinary care than non- brachycephalic dogs [34]. In addition, we identified that FCI group 2 breeds required the highest average number of veterinary care events. This group includes the larger molossoid dog breeds which others have previously identified as having higher mortality [32, 44]. The primitive FCI group 5 breeds had the lowest average morbidity of all the groups, which has not been reported previously, except for the Norrbottenspitz breed [45]. This may be, in part, due to the large number of primitive breeds for which there is insurance data available in our data set, while other studies may not have had health data available for these breeds.

There were interesting exceptions to the correlation of inbreeding and health. The Border terrier, Basenji, Collie, and English setter breeds have high inbreeding but low morbidity. Likewise, the Malinois, Pomeranian and Russian Tsvetnaya Bolonka (Russian Toy) have lower inbreeding and high morbidity. These example breeds are neither brachycephalic nor particularly known for extreme morphologies. In the case of healthy breeds with high inbreeding, it may be possible that these breeds have been purged of deleterious alleles as has happened with inbred mouse strains [46]. In the opposite situation (lower inbreeding and high morbidity), the recorded morbidities could be high allele frequency Mendelian diseases or potentially conditions linked to phenotypes under selection in the breed. These discrepancies could also exist due to population differences between the insurance data and the inbreeding data.

A caveat for this analysis is the utilization of breed average values rather than specific measurements on individual animals for body weight, inbreeding and health. The utilization of breed average values could lead to over or under estimates of the effects of inbreeding on health. As veterinary health databases become more developed and DNA testing more widespread this type of data would be extremely useful across large numbers of individual dogs. None the less, inbreeding values from this work were comparable to levels obtained by other researchers using different methodologies and different populations of dogs [8, 9, 47, 48].

A demonstration of direct negative effects of inbreeding within breeds has been limited, likely due to the need to use molecular tools to determine actual historic inbreeding, the absence of dogs with low inbreeding (< 0.1) or no inbreeding and the challenges of phenotyping for individual animals. Inbreeding (range 0.2–0.48) based on runs of homozygosity was associated with smaller litters in Golden retrievers [49]. Inbreeding in dams (0.32–0.45) was associated with fertility in the Entlebucher Mountain dog [50]. One challenge for the associations performed within breeds is the overall high level of inbreeding within the breeds which does not allow the full range of inbreeding levels to be evaluated relative to health traits. In this study we took advantage of the availability of insurance data to evaluate morbidity. It is estimated that 40% of dogs in Sweden are insured, allowing the insurance data to provide a good estimate of the population as a whole [51], and the ability to use dogs years at risk in the denominator allows comparisons across breeds and factors in length of insurance coverage [31, 52]. One challenge with comparing across breeds is that insurance coverage is ended for breeds at different ages and the average age of insured dogs differs between breeds.

The heterozygosity values were obtained from worldwide sample collection centered in the Scandinavian countries. Dataset 2 was from samples collected predominantly in the United States and there was strong correlation with the heterozygosity values. Other groups have also identified strong correlation between heterozygosity values and inbreeding based on commercial testing in the United States [9]. The insurance data is mostly from Sweden, however Agria Pet Insurance does operate in other countries as well. There is some evidence of population stratification between countries for different breeds and types of dogs, however the effect on overall levels of inbreeding was not high [53].

The results for the effects of inbreeding on mortality have been varied between studies with some finding a significant effect [32] and others not [9]. One reason that previous results were inconsistent with respect to the effects of inbreeding on mortality may be the consistently high levels of inbreeding that exist across the majority of purebred dog breeds such that there was not enough variation in the levels of inbreeding to detect an effect.

One must consider that the majority of dog breeds displayed high levels of inbreeding well above what would be considered safe for either humans or wild animal populations. The effects of inbreeding on overall fitness have been demonstrated experimentally using mice, where an overall reduction in fitness between mice with F = 0.25 compared to F = 0 was determined to be 57% [54]. While this high level of inbreeding was less relevant to many captive and wild species, it is highly relevant to purebred dogs, based on the average inbreeding identified in this study. However the rate of inbreeding between these mouse experiments and what has occurred in dogs breeds is not the same and could have an effect on health. In humans, modest levels of inbreeding (3–6%) were shown to be associated with increased prevalence of late onset complex diseases [55] as well as other types of inbreeding depression [11]. These findings in other species combined with the incredibly strong breed predispositions to complex diseases like cancers and autoimmune diseases highlight the potential relevance of high inbreeding in dogs to their health.

## Conclusions

Additional studies to evaluate the effects of inbreeding on health need to be performed on an individual animal basis and should include the entire scale of inbreeding levels. Careful management of breeding populations to avoid additional loss of existing genetic diversity, through breeder education and monitoring of inbreeding levels enabled by direct genotyping technologies, is essential. Outcrosses are being proposed for some breeds and conditions [56, 57] or have already been carried out as a measure to increase genetic diversity. Care must be taken to consider if these will effectively increase overall breed diversity and therefore reduce inbreeding [58]. In particular, in the few breeds with low inbreeding levels, every effort should be made to maintain the genetic diversity that is present. While history has shown how easily it can be lost in closed breed populations, encouragement can be found in breeds exemplifying a consistent and true-breeding type without high inbreeding levels.

## Methods

### Samples

Dataset 1 (Additional file 1). Median SNP heterozygosity values were obtained for 227 breeds or varieties based on 30–2520 individuals per breed (Total Count = 49,378) using a genetic diversity screening test commercially available as MyDogDNA™ or Optimal Selection™ (Wisdom Health, Vancouver, WA, USA). This dataset consisted of non-invasive cheek swab samples collected by dog owners, and either blood or cheek swab samples collected at certified veterinary clinics, for submission to commercial DNA testing. The samples were submitted for MyDogDNA™ / Optimal Selection™ analysis between April 3rd, 2015 and June 23th, 2020. The breed of a dog was reported by its owner, typically with additional accompanying information confirming registration under Fédération Cynologique Internationale (FCI), American Kennel Club (AKC), United Kennel Club (UKC), or the Kennel Club (UK)

Dataset 2 (Additional file 2). DNA samples from 274 dogs (19 breed groups) were collected as controls for disease studies (Additional file 2). Dogs were selected that were unrelated to the second generation based on pedigree where available. Eight to twenty individuals per breed were used to determine F (see below). Fifty-nine dogs designated by their owners as mixes were also used in two groups: 33 were used to establish a SNP list for analysis (see F below) and the remaining 26 were used in the analysis as mixed breed samples

### Agria insurance data

Agria Insurance data in the form of breed profiles were accessed from the International Partnership for Dogs (dogwellnet.com) and the data abstracted for use in this study. Agria insurance morbidity in veterinary care events/10,000 dog years at risk (DYAR) from 2011 to 2016 account for the actual time the dog was insured. Individuals were assessed only once for each category/diagnosis for > = 1 veterinary care event (VCE). Morbidity values are the number of dogs experiencing VCE within each category/diagnosis that were not for preventive medicine or prophylactic measures since these are not covered by the policy. Agria insurance data was available for 162 breeds and mixes with some breed varieties combined differently from the Heterozygosity values of dataset 1 (Additional file 1).

Categorization as brachycephalic was determined for each breed based on breed standards as shown in Additional file 1. Group designations were based on the FCI group divisions with the exception of the dachshunds which were grouped as hounds in FCI group 6. Mid-range body weight for all breeds was obtained from the low female and high male weights if there were sex differences or between the low and high weight if there were no sex differences listed (www.akc.org).

### Inbreeding coefficient (F)

All dogs from Dataset 2 were genotyped on the Illumina canine HD genotyping array (173 K SNPs). Plink [59] was used to prune SNPs in 33 mixed breed dogs to identify SNPs in linkage equilibrium using the following parameters: --indep-pairwise 200, 50, 0.6, <window size 200 kb < step size [50]> < *r*^*2*^ 0.6. This resulted in 96,497 autosomal markers. F as reported in this manuscript was calculated using the “ibc” function and FHAT 2 as implemented in plink which is based on the program GCTA (<observed hom. Count> − <expected count>) / (<total observations> − <expected count>).

### Adjusted inbreeding coefficient (F_adj)_

F from dataset 2 was highly correlated with H from dataset 1 (Pearson correlation coefficient of − 0.8899 (*P* < 0.0001) for the same 19 breeds. Linear regression was performed to obtain adjusted F values for each breed (Y = − 0.02103*X + 0.9456, *r*^*2*^ = 0.79). The H values were adjusted based on the regression to provide breed estimates of inbreeding for 227 breeds (F_adj_).

### Statistics

Pearson Correlation, descriptive statistics, Mann Whitney *U* test, Kruskal-Wallis and linear regression as indicated were performed in GraphPad Prism (V. 9.0.1). Spearman correlation was performed for correlation of Morbidity and F_adj_ and Morbidity and Body size since they were not normally distributed.

### Multiple regression analysis

Modelling the morbidity scores as a function of the breed body weight and mean breed F_adj_ considers this simple linear model:

1 $${y}_i={b}_0+{b}_w{Weight}_i+{b}_f{F}_i+{e}_i$$

where *y*_*i*_ is the observed morbidity score for the *i*-th breed, *b*_0_ is an unknown constant common to all breeds, *b*_*w*_ is the regression coefficient of mid-range body weight of the *i*-th breed (measured as *Weight*_*i*_) on morbidity, *b*_*f*_ is the regression coefficient for average adjusted F of the *i*-th breed (measured as *F*_*i*_ on a scale from 0 to 1.0) on morbidity, and *e*_*i*_ is the unobserved residual error for the *i*-th breed. We consider the residual term for the *i*-th breed (i.e., *e*_*i*_) to have null mean and variance ${\sigma}_e^2/{n}_i$, where *n*_*i*_ is the sample size for the *i*-th breed.

An additional, and necessary, prerequisite of this analysis is the recognition that the variation in breed morbidity changes with breed body weight, there being more variability “in the tails” of this distribution than nearer the median. This form of heteroscedasticity can be better accommodated through the application of robust regression models [60]. Accordingly, model [1], was evaluated in the MASS package [61] of the public domain statistical language R [62] . Tests of hypotheses regarding estimates of model parameters were facilitated through the package sfsmisc [63]. *r*^*2*^ was computed (the 0.77 value) recognizing the fact that data points were weighted by sample size.

## Supplementary Information

**Additional file 1: Dataset 1.** Table of breeds, numbers of individuals, heterozygosity values, F_adj_ values, morbidity, group and morphology classifications.

**Additional file 2: Dataset 2.** Breeds used for linear regression. Numbers of individuals, median, minimum and maximum inbreeding and median, minimum and maximum heterozygosity values are included.

## Data Availability

All data generated or analyzed during this study are included in this published article and its additional files.
